# Proteolytic Shedding of Human Colony‐Stimulating Factor 1 Receptor and its implication

**DOI:** 10.1111/jcmm.16474

**Published:** 2021-03-30

**Authors:** Yue Wei, Menghui Ma, Sheng Lin, Xin Li, Yue Shu, Ziwei Wang, Yuhang Zhou, Banglian Hu, Baoying Cheng, Shengshun Duan, Xiaohua Huang, Huaxi Xu, Yun‐Wu Zhang, Honghua Zheng

**Affiliations:** ^1^ Fujian Provincial Key Laboratory of Neurodegenerative Disease and Aging Research Institute of Neuroscience School of Medicine Xiamen University Xiamen China; ^2^ Basic Medical Sciences School of Medicine Xiamen University Xiamen China; ^3^ Shenzhen Research Institute Xiamen University Shenzhen China

**Keywords:** CSF1R, CSF1R I794T variant, proteolytic shedding, TREM2

## Abstract

Both Colony‐stimulating factor 1 receptor (CSF1R) and triggering receptor expressed on myeloid cells‐2 (TREM2) are trans‐membrane receptors and are expressed in the brain primarily by microglia. Mutations in these two microglia‐expressed genes associated with neurodegenerative disease have recently been grouped under the term “microgliopathy”. Several literatures have indicated that CSF1R and TREM2 encounters a stepwise shedding and TREM2 variants impair or accelerate the processing. However, whether CSF1R variant affects the shedding of CSF1R remains elusive. Here, plasmids containing human CSF1R or TREM2 were transiently transfected into the human embryonic kidney (HEK) 293T cells. Using Western Blot and/or ELISA assay, we demonstrated that, similar to those of TREM2, an N‐terminal fragment (NTF) shedding of CSF1R ectodomain and a subsequent C‐terminal fragment (CTF) of CSF1R intra‐membrane were generated by a disintegrin and metalloprotease (ADAM) family member and by γ‐secretase, respectively. And the shedding was inhibited by treatment with Batimastat, an ADAM inhibitor, or DAPT or compound E, a γ‐secretase inhibitor. Importantly, we show that the cleaved fragments, both extracellular domain and intracellular domain of a common disease associated I794T variant, were decreased significantly. Together, our studies demonstrate a stepwise approach of human CSF1R cleavage and contribute to understand the pathogenicity of CSF1R I794T variant in adult‐onset leukoencephalopathy with axonal spheroids and pigmented glia (ALSP). These studies also suggest that the cleaved ectodomain fragment released from CSF1R may be proposed as a diagnostic biomarker for ALSP.

## 1 INTRODUCTION

Colony‐stimulating factor 1 receptor (CSF1R), a single trans‐membrane receptor containing a ligand‐binding domain in the extracellular region and a tyrosine kinase domain in the cytoplasm, is predominantly expressed in monocyte, macrophage and bone marrow cell precursors, as well as microglia in central nervous system (CNS).^1^, ^2^ Studies have revealed that people with *CSF1R* variants bear an adult‐onset neurodegenerative disorder, named adult‐onset leukoencephalopathy with axonal spheroids and pigmented glia (ALSP).^2^, ^3^ Furthermore, genetic variants of triggering receptor expressed on myeloid cells 2 (TREM2), a trans‐membrane receptor specifically expressed in microglia in the brain, increase the risk of developing neurodegenerative disorders including Alzheimer's disease (AD).^4^ Studies have shown that TREM2 undergoes a proteolytic processing.^5^ T66 M or Y38C mutation in the immunoglobulin (Ig)‐like extracellular domain of TREM2 results in reduced shedding ^6^ whereas TREM2 H157Y variant leads to enhanced proteolysis.^7^ CSF1R has been reported to be involved in two separate cleavage events.^8^, ^9^ As a pathogenic variant for ALSP, I794T is the most common variant of CSF1R among the identified seventy more variants.^2^ However, whether CSF1R undergoes a proteolytic cleavage similar to that of TREM2 and whether CSF1R variant affects the proteolytic shedding of CSF1R fragments remain largely elusive.

This study demonstrates a two‐step proteolytic processing of CSF1R and the decreased cleavage fragments of disease‐associated CSF1R I794T variant, indicating that the extracellular fragment of CSF1R may be a potential biomarker for the early diagnosis of ALSP.

## 2 MATERIALS AND METHODS

### 2.1 Reagents and Antibodies

Batimastat and compound E were purchased from Millipore. DAPT was bought from Sigma‐Aldrich. Cycloheximide (CHX) was from APExBIO and CSF1 was from R&D Systems. The following antibodies, GFP Tag antibody (RRID: AB_11182611) and HA Tag antibody (RRID: AB_1104232), were from Proteintech. Anti‐MCSF receptor antibody (peptide selected from 955‐972; RRID: AB_776253) was from Abcam and c‐Fms/CSF‐1R antibody (B‐8) (peptide selected from 11‐310; RRID: AB_627620) from Santa Cruz. Phospho‐CSF1R (Tyr723) (49C10) rabbit mAb was bought from Cell Signaling Technology. Goat anti‐mouse or anti‐rabbit IgG (H + L) secondary antibodies were all from Thermo (RRID: AB_228307; RRID: AB_228341).

### 2.2 cDNA Constructs

**FIGURE 1:**
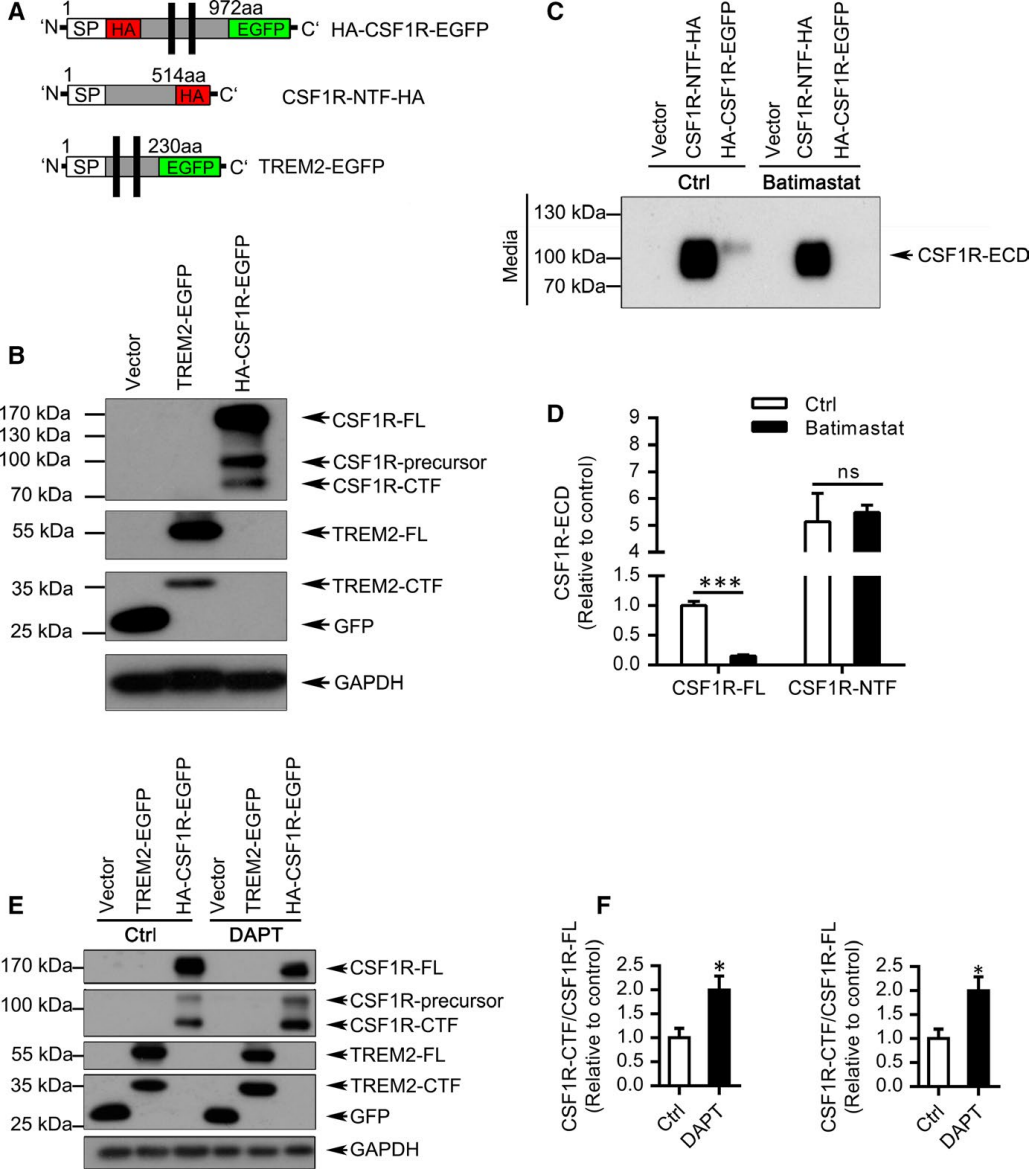
Cleaved fragments of CSF1R were detected in cell lysate and the medium. A, Sketch of the full length of CSF1R, N‐terminal fragment of CSF1R and full length of TREM2 plasmid. B, HEK293T cells were transfected with pEGFP‐N1 (Vector), TREM2‐EGFP or HA‐CSF1R‐EGFP. After 24 hours, cells were harvested and different fragments of CSF1R were detected by Western blotting with anti‐GFP antibody. The CSF1R‐precursor (~110 kDa), the full length of CSF1R (CSF1R‐FL, ~170 kDa) and the C‐terminal fragment of CSF1R (CSF1R‐CTF, ~ 75 kDa) were detected for CSF1R in cell lysate represent different fractions. Vector is a negative control and TREM2‐EGFP is a positive control. C, HEK293T cells transfected with pEGFP‐N1 (Vector), CSF1R‐NTF‐HA or HA‐CSF1R‐EGFP were cultured with or without 10 μM Batimastat in serum‐free DMEM for 16 hours. The extracellular domain of CSF1R (CSF1R‐ECD) was detected by Western blotting with anti‐HA antibody. D, *Bar charts* show the relative levels of CSF1R‐ECD (n = 3). CSF1R‐ECD is significantly reduced in the Batimastat group when compared to that in control group. Vector is the negative control and CSF1R‐NTF‐HA is a positive control (n = 3). ***, *P* <.001, ns, not significant. E, HEK293T cells transfected with pEGFP‐N1 (Vector), TREM2‐EGFP or HA‐CSF1R‐EGFP were incubated with or without 10 μM DAPT for 16 hours. The full‐length and C‐terminal fragments of CSF1R and TREM2 were detected by Western blotting with anti‐GFP antibody. F, Error bars indicate SEM of at least three independent experiments. The CSF1R‐CTF was notably increased in the DAPT group compared to that in control group. Vector is a negative control, and TREM2‐EGFP is a positive control (n ≥ 3). Statistical analysis was done by *t* test. *, *P* <.05

The vectors used in these experiments are as follows: TREM2‐GFP, pEGFP‐N1, HA‐CSF1R‐EGFP, pCMV3.1‐HA, CSF1R‐NTF‐HA, CSF1R‐HA, CSF1R‐I794T‐HA (Figure 1A). The full‐length CSF1R in HA‐CSF1R‐EGFP was obtained from the 972 amino acid of CSF1R in pCMV3‐CSF1R‐HA (Sino Biological, Beijing, China). Plasmid extraction was performed after sequencing verification.

### 2.3 Cell Culture and Treatments

HEK 293T cells were cultured in DMEM containing 10% FBS and were transfected with different plasmids using TurboFect transfection reagent (Thermo) according to the manufacturer's specifications. Transfected cells were incubated with or without 10 μM Batimastat, 500 nM compound E or 10 μM DAPT for 16 hours. Cells or medium were then collected for the following Western blotting and/or ELISA assay.

### 2.4 Western Blot

Equivalent protein samples or supernatant samples were tested by SDS‐PAGE and transferred to a PVDF membrane. Proteins bound with primary (1:1000 CSF1R or 1:2000 GFP or 1:10 000 HA) and secondary antibodies were visualized by ECL and analysed by Image J software.

### 2.5 ELISA assay

The CSF1R‐NTF in the conditioned medium was tested using a microplate reader (Thermo, USA) at 450 nm according to the instructions of Human CSF1R ELISA Kit (Sino Biological, Beijing).

### 2.6 Statistical analysis

Data were represented as mean ± SEM. Statistical significance was determined using two‐way analysis of variance (ANOVA) test (GraphPad Prism 7.0) or two‐tailed Student's *t* test. A *p* value less than 0.05 was considered significant.

## 3 RESULTS

### 3.1 Sequential Shedding of the CSF1R by ADAM family and γ‐secretase

We first analysed whether CSF1R suffers proteolytic shedding at the cell surface. As a positive control, the shedding of TREM2‐EGFP was detected simultaneously by Western blotting (Figure 1A‐B). Robust levels of CSF1R‐precursor, the full length of CSF1R (CSF1R‐FL), and the C‐terminal fragment of CSF1R (CSF1R‐CTF) were detected (Figure 1B).

Given that members of the disintegrin and metalloprotease (ADAM) family are major proteases involved in the proteolytic processing of cell surface proteins, HEK293T cells transfected with CSF1R N‐terminal fragment (CSF1R‐NTF) or HA‐CSF1R‐EGFP were incubated with or without Batimastat, a known inhibitor of ADAM families.^10^ Interestingly, Batimastat treatment resulted in a dramatical decrease of CSF1R ectodomain (CSF1R‐ECD) in the medium (Figure 1C‐D) and the accumulation of CSF1R‐FL (Figure S1A‐D) in cell lysates, indicating that the ectodomain of CSF1R can be shed by ADAM and this cleaved CSF1R‐ECD can be released into the medium. Previous studies suggested that C terminus of proteins usually selectively processed by γ‐secretase.^11^ Intriguingly, treatment with DAPT, a γ‐secretase inhibitor, resulted in a dramatic increase of CSF1R‐CTF, which is consistent with the inhibition effects of γ‐secretase on TREM2‐CTF accumulation (Figure 1E‐F). These findings were further confirmed by another γ‐secretase inhibitor compound E in which both CSF1R‐CTF and TREM2‐CTF were notably accumulated (Figure S2A‐C).

### 3.2 Decreased cleavage fragments of disease‐associated CSF1R‐I794T variant

**FIGURE 2:**
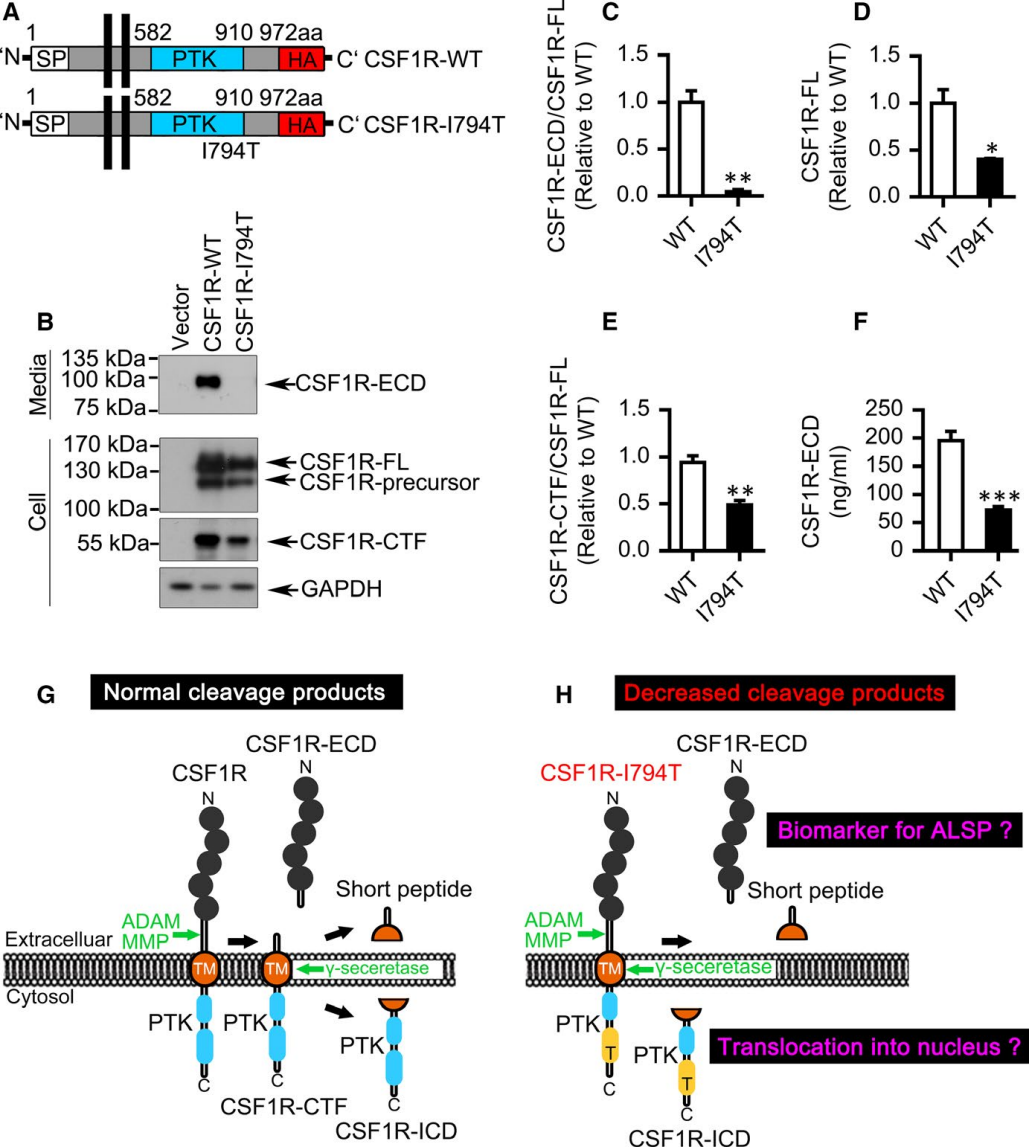
Decreased cleavage fragments of disease‐associated CSF1R I794T variant. A, Sketch of the wild‐type (WT) of CSF1R and CSF1R I794T mutant plasmid. B, HEK293T cells were transfected with pCMV3.1‐HA (Vector), CSF1R‐HA (CSF1R‐WT) or CSF1R‐I794T‐HA. Cell lysates were collected and detected by Western blotting with anti‐CSF1R (epitope 11 aa ~ 310 aa; epitope 955 aa ~ 972 aa) or HA‐tag antibody. C, A dramatically reduction of the ECD ratio to the full length (FL) of CSF1R I794T in the medium detected by Western blotting. D, The FL of CSF1R I794T was significantly decreased compared to that of WT CSF1R. E, The ratio of CTF to FL of CSF1R I794T was significantly decreased compared to that of WT CSF1R. F, A dramatically reduction of the secreted ECD of CSF1R I794T in the medium detected by ELISA assay. G, Schematic shedding of CSF1R. CSF1R undergoes a proteolytic cleavage similar to that of TREM2. Extracellular of the CSF1R ectodomain can be shed by MMP or ADAM family and the intramembrane of the CSF1R C‐terminal fragment can be subsequently cleaved by γ‐secretase. H, Schematic shedding of CSF1R I794T variant. The cleaved fragments of disease‐associated CSF1R I794T, underlying the white matter disease of ALSP, were significantly decreased. The released CSF1R ectodomain (ECD) may be proposed as a diagnostic biomarker for ALSP. Whether the released CSF1R intercellular domain (ICD) translocates into the nucleus remains elusive. *Error bars* indicate SEM of at least three independent experiments. Statistical analysis was done by *t* test (n = 3). *, *P* <.05, **, *P* <.01, ***, *P* <.001

The common mutation CSF1R I794T affects the tyrosine–kinase activity of CSF1R causing ALSP.^2^ Compared with wild‐type (WT) CSF1R, the level of CSF1R‐ECD in the CSF1R I794T mutant culture medium was almost undetectable quantified by Western blotting and ELISA assay (Figure 2A‐F). Moreover, the expression of CSF1R‐ECD or CSF1R‐CTF in CSF1R I794T mutant cells was all dramatically reduced, though the full length of CSF1R I794T was also significantly decreased (Figure 2A‐F). Unexpectedly, we found that I794T variant did not affect the transcription (Figure S3A) or the stability of CSF1R (Figure S3B‐D). Furthermore, the I794T variant cannot be phosphorylated (Y723) in response to CSF1, the ligand of CSF1R (Figure S4A‐B). These results indicate the dysfunction of ALSP‐associated I794T variant.

## 4 DISCUSSION

Together, we show that CSF1R undergoes a two‐step proteolytic cleavage similar to that of TREM2. Extracellular of the CSF1R ectodomain can be shed by ADAM family and the intramembrane of the CSF1R‐CTF can be subsequently cleaved and generated by γ‐secretase. Notably, we can detect CSF1R‐ECD in the wild‐type CSF1R medium whereas CSF1R‐ECD from CSF1R‐I794T mutant was almost undetectable. Thus, the present data demonstrate a stepwise approach of human CSF1R cleavage and that CSF1R fragments may execute important physiological and pathophysiological functions (Figure 2G‐H).

Given that the soluble form of CSF1R (sCSF1R) was detected in goldfish serum ^12^ and that soluble TREM2 can be detected in human cerebrospinal fluid,^13^ whether sCSF1R, namely CSF1R‐ECD, can be determined in human cerebrospinal fluid or serum of ALSP patients as a biomarker needs to be explored. Whether sCSF1R plays a role in CNS, similar to the function of soluble TREM2 in microglial activation, remains elusive.^14^

It has been proposed that the cytoplasmic domain of CSF1R can translocate to the nucleus.^8^ Thus, it will be interesting to investigate whether the nuclear translocation of CSF1R‐ICD regulates genes expression. Moreover, due to the dramatically reduced expression of CSF1R‐CTF released from CSF1R I794T mutant, it will be interesting to further investigate whether this reduction is a result of the degradable nature of CSF1R I794T mutant or the abnormal shedding of the ALSP‐associated I794T variant. Although previous studies showed that I794T and other CSF1R mutant proteins expressed in a factor‐dependent cell line are exposed normally on the cell surface,^3^, ^15^ this dispute needs to be clarified. Of course, additional experiments, such as samples from CSF1R variant bearing human brain or mutant knock‐in animal models, are needed to be performed to see how this variant contributes to the biology of ALSP.

## CONFLICT OF INTEREST

The authors have no conflict of interest to declare.

## AUTHOR CONTRIBUTION

**Yue Wei:** Data curation (equal); Methodology (equal); Software (equal); Writing‐original draft (equal). **Menghui Ma:** Data curation (equal); Methodology (equal); Software (equal); Writing‐review & editing (equal). **Sheng Lin:** Data curation (equal); Methodology (equal); Software (equal); Writing‐review & editing (equal). **Xin Li:** Data curation (equal); Methodology (equal); Software (equal); Writing‐review & editing (equal). **Yue Shu:** Data curation (supporting); Methodology (supporting); Resources (equal); Software (supporting); Validation (supporting); Visualization (supporting); Writing‐review & editing (supporting). **Ziwei Wang:** Data curation (equal); Methodology (supporting); Software (supporting); Validation (supporting); Writing‐review & editing (supporting). **Yuhang Zhou:** Data curation (supporting); Methodology (supporting); Validation (supporting); Writing‐review & editing (supporting). **Banglian Hu:** Data curation (supporting); Methodology (supporting); Validation (supporting). **Baoying Cheng:** Resources (supporting); Visualization (supporting). **Shengshun Duan:** Methodology (supporting); Resources (supporting). **Xiaohua Huang:** Project administration (supporting). **Huaxi Xu:** Project administration (supporting); Supervision (supporting); Writing‐review & editing (supporting). **Yun‐Wu Zhang:** Funding acquisition (equal); Project administration (supporting); Supervision (supporting); Writing‐review & editing (supporting). **Honghua Zheng:** Data curation (supporting); Funding acquisition (lead); Methodology (supporting); Project administration (lead); Software (supporting); Supervision (lead); Writing‐original draft (lead); Writing‐review & editing (lead).

## Supporting information

Supporting information

## Data Availability

The authors are willing to provide the data related to this manuscript upon reasonable request.

## References

[1] Chitu V , Gokhan S , Nandi S , Mehler MF , Stanley ER . Emerging roles for csf‐1 receptor and its ligands in the nervous system. Trends Neurosci. 2016;39(6):378‐393. 27083478 10.1016/j.tins.2016.03.005 PMC4884457

[2] Konno T , Kasanuki K , Ikeuchi T , Dickson DW , Wszolek ZK . CSF1R‐related leukoencephalopathy: A major player in primary microgliopathies. Neurology. 2018;91(24):1092‐1104. 30429277 10.1212/WNL.0000000000006642 PMC6329328

[3] Hume DA , Caruso M , Ferrari‐Cestari M , Summers KM , Pridans C , Irvine KM . Phenotypic impacts of CSF1R deficiencies in humans and model organisms. J Leukoc Biol. 2020;107(2):205‐219. 31330095 10.1002/JLB.MR0519-143R

[4] Zheng H , Cheng B , Li Y , Li X , Chen X , Zhang YW . TREM2 in Alzheimer's disease: microglial survival and energy metabolism. Front Aging Neurosci. 2018;10:395. 30532704 10.3389/fnagi.2018.00395 PMC6265312

[5] Wunderlich P , Glebov K , Kemmerling N , Tien NT , Neumann H , Walter J . Sequential proteolytic processing of the triggering receptor expressed on myeloid cells‐2 (TREM2) protein by ectodomain shedding and gamma‐secretase‐dependent intramembranous cleavage. J Biol Chem. 2013;288(46):33027‐33036. 24078628 10.1074/jbc.M113.517540 PMC3829152

[6] Kleinberger G , Yamanishi Y , Suarez‐Calvet M , et al. TREM2 mutations implicated in neurodegeneration impair cell surface transport and phagocytosis. Sci Transl Med. 2014;6(243):243ra286. 10.1126/scitranslmed.3009093 24990881

[7] Thornton P , Sevalle J , Deery MJ , et al. TREM2 shedding by cleavage at the H157–S158 bond is accelerated for the Alzheimer's disease‐associated H157Y variant. EMBO Mol Med. 2017;9(10):1366‐1378. 28855301 10.15252/emmm.201707673 PMC5623839

[8] Wilhelmsen K , van der Geer P . Phorbol 12‐myristate 13‐acetate‐induced release of the colony‐stimulating factor 1 receptor cytoplasmic domain into the cytosol involves two separate cleavage events. Mol Cell Biol. 2004;24(1):454‐464. 14673177 10.1128/MCB.24.1.454-464.2004 PMC303356

[9] Vahidi A , Glenn G , van der Geer P . Identification and mutagenesis of the TACE and γ‐secretase cleavage sites in the colony‐stimulating factor 1 receptor. Biochem Biophys Res Commun. 2014;450(1):782‐787. 24955855 10.1016/j.bbrc.2014.06.061

[10] Lambrecht BN , Vanderkerken M , Hammad H . The emerging role of ADAM metalloproteinases in immunity. Nat Rev Immunol. 2018;18(12):745‐758. 30242265 10.1038/s41577-018-0068-5

[11] Hemming ML , Elias JE , Gygi SP , Selkoe DJ . Proteomic profiling of gamma‐secretase substrates and mapping of substrate requirements. PLoS Biol. 2008;6(10):e257. 18942891 10.1371/journal.pbio.0060257 PMC2570425

[12] Barreda DR , Hanington PC , Stafford JL , Belosevic M . A novel soluble form of the CSF‐1 receptor inhibits proliferation of self‐renewing macrophages of goldfish (Carassius auratus L.). Dev Comp Immunol. 2005;29(10):879‐894. 15978283 10.1016/j.dci.2005.02.006

[13] Piccio L , Buonsanti C , Cella M , et al. Identification of soluble TREM‐2 in the cerebrospinal fluid and its association with multiple sclerosis and CNS inflammation. Brain. 2008;131(Pt 11):3081‐3091. 18790823 10.1093/brain/awn217 PMC2577803

[14] Zhong L , Chen XF , Wang T , et al. Soluble TREM2 induces inflammatory responses and enhances microglial survival. J Exp Med. 2017;214(3):597‐607. 28209725 10.1084/jem.20160844 PMC5339672

[15] Pridans C , Sauter KA , Baer K , Kissel H , Hume DA . CSF1R mutations in hereditary diffuse leukoencephalopathy with spheroids are loss of function. Sci Rep. 2013;3:3013. 24145216 10.1038/srep03013 PMC3804858

