# Supplementary material for: Proteolytic Shedding of Human Colony‐Stimulating Factor 1 Receptor and its implication
Source: J Cell Mol Med. 2021 Mar 30;25(9):4516–21. doi: 10.1111/jcmm.16474 (PMC8093967; doi:10.1111/jcmm.16474)
Supplement: Supplementary file 1 — Supporting information [file JCMM-25-4516-s001.docx]

**Reverse transcription (RT) and quantitative polymerase chain reaction (qPCR)**—Total RNA was isolated from 293T cells using TRIzol reagent (CWBIO). Reverse transcription was performed using HiFiScript gDNA Removal RT MasterMix (CWBIO) and the resulting cDNA was used for qRT-PCR. The set of *ACTB* primers was used as an internal control for each specific gene amplification. The relative levels of expression were quantified and analyzed by using Roche LightCycler480 software. The real-time value for each averaged sample was compared and normalized against control levels. The primers sequences used to amplify target genes *CSF1R* and *ACTB* were listed in supplementary Table 1.

**Supplementary Table1 Primers sequences used to amplify target genes.**

| Gene | Primer |
| --- | --- |
| *homo* *CSF1R*-forward | 5’-CAGAGCCTGCTGACTGTTGA-3’ |
| *homo* *CSF1R*-reverse | 5’- GGACCTGGTACTTGGGCTTC-3’ |
| *homo* *ACTB*-forward | 5’- ATCAAGATCATTGCTCCTCCTGAG -3’ |
| *homo* *ACTB*-reverse | 5’- CTGCTTGCTGATCCACATCTG-3’ |

**
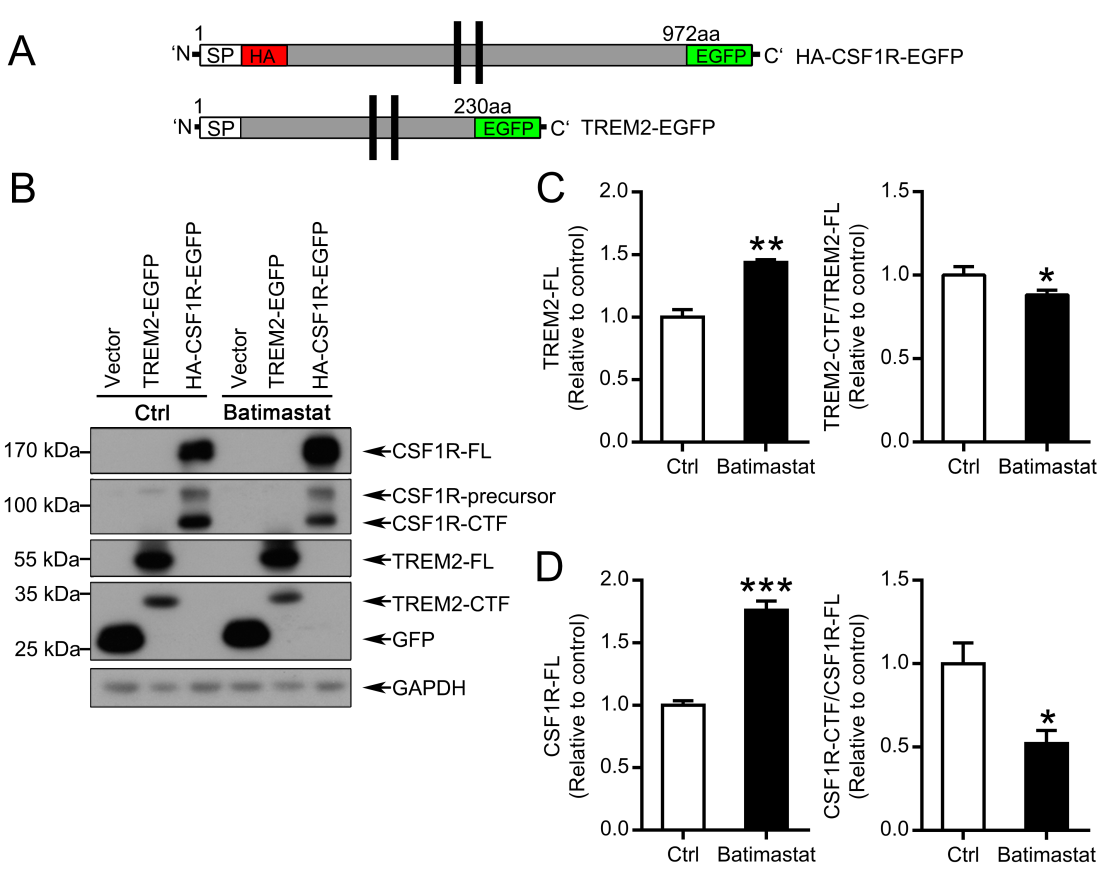
**

**Supplementary Figure 1.** **Accumulation of CSF1R-FL** **in response to ADAM inhibitor Batimastat**

**A**. Sketch of the full-length of CSF1R and TREM2 plasmid. **B**. HEK293T cells transfected with pEGFP-N1 (Vector), TREM2-EGFP or HA-CSF1R-EGFP were incubated with or without 10 μM Batimastat for 16 hours. The full-length and C-terminal fragments of CSF1R and TREM2 were detected by Western blotting with anti-GFP, anti-HA-tag or anti-CSF1R antibody. **C**. The TREM2-FL was significantly increased and the TREM2-CTF was notably reduced in the Batimastat group compared to that in control group. **D**. The CSF1R-FL was significantly increased and the CSF1R-CTF was notably reduced in the Batimastat group compared to that in control group. Error bars indicate SEM of at least three independent experiments. Vector is used as a negative control and TREM2-EGFP is used as a positive control (n = 3). Statistical analysis was done by *t* test. *, *p* < 0.05, **, *p* < 0.01 , ***, *p* < 0.001.

**
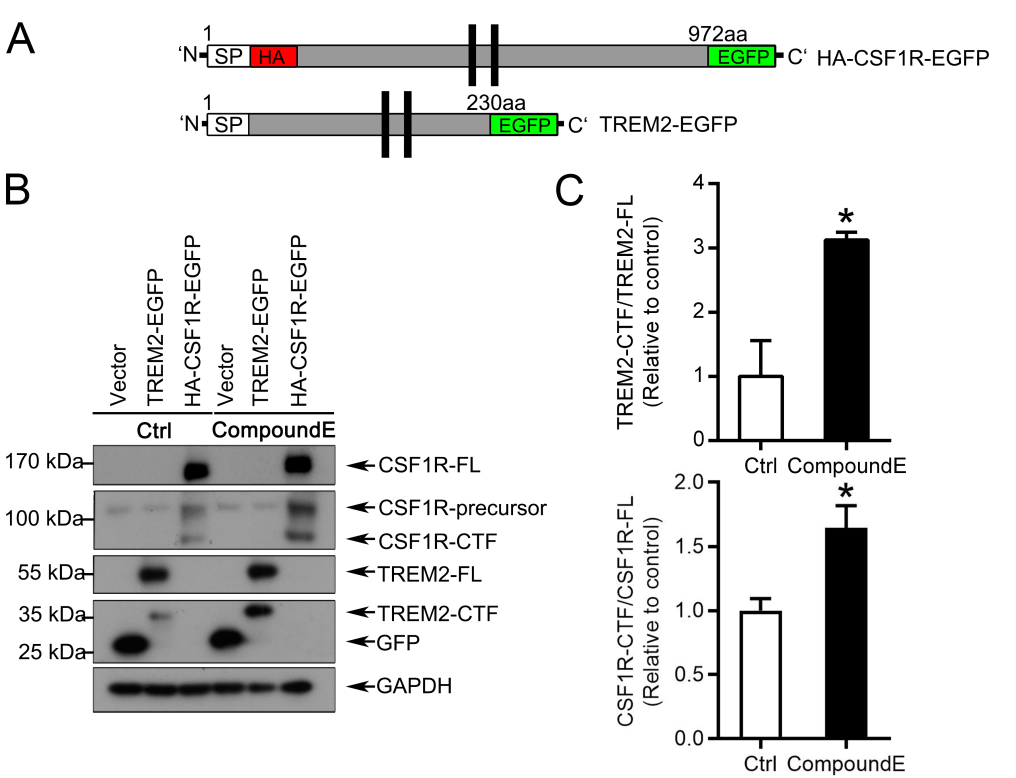
**

**Supplementary Figure 2.** **CSF1R-CTF was accumulated in response to γ-secretase inhibitor Compound E**

**A**. Sketch of the full-length of CSF1R and TREM2 plasmid. **B**. HEK293T cells transfected with pEGFP-N1 (Vector), TREM2-EGFP or HA-CSF1R-EGFP were incubated with or without 500 nM Compound E for 16 hours. The full-length and C-terminal fragments of CSF1R and TREM2 were detected by Western blotting with anti-GFP antibody. **C**. *Error bars* indicate SEM of at least three independent experiments. The CSF1R-CTF was notably accumulated in the Compound E group compared to that in control group. Vector is a negative control and TREM2-EGFP is a positive control (n ≥ 3). Statistical analysis was done by *t-*test. *, *p* < 0.05.

**
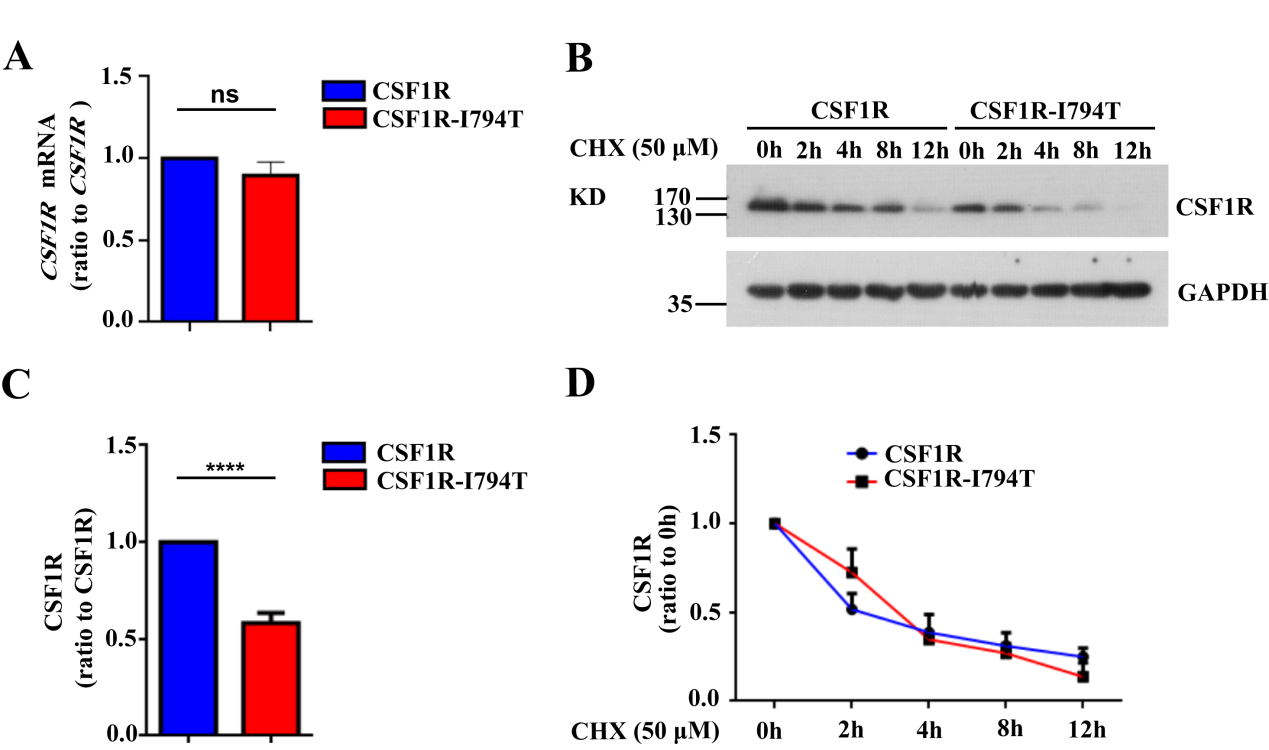
**

**Supplementary Figure 3. I794T variant does not affect the transcription or stability of CSF1R.** For the protein stability experiments, HEK 293T cells were transfected with CSF1R or p.I794T mutant plasmid following 50 μM CHX treatments for 0, 2, 4, 8 or 12 hrs. **A.** The mRNA level of CSF1R was measured by RT-qPCR (n=3). ns, not significant. **B.** The transfected cells were further treated with CHX for indicated times. Protein levels in cell lysates from those cells were analyzed by Western blotting. Representative Western blots of total CSF1R are shown. **C.** The protein expression of CSF1R was significantly decreased in HEK 293T cells transfected with CSF1R-I794T mutation compared to wild type CSF1R (n=3). ****, *p* < 0.000. **D.** Upon CHX treatment, the stability of total CSF1R between wild type CSF1R or CSFIR-I794T groups were not significantly changed (n=3).


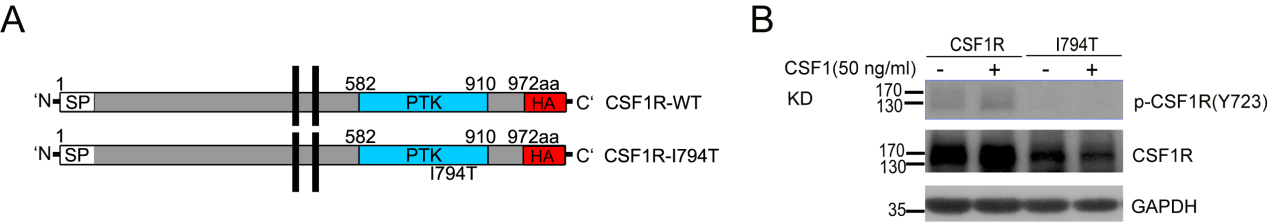


**Supplementary Figure 4. The phosphorylation of wild-type of CSF1R but not CSFIR-I794T variant was activated upon CSF1 treatment.** HEK 293T cells were transfected with wild type CSF1R or CSFIR-I794T plasmids. **A**. Sketch of the wild-type of CSF1R and CSF1R I794T mutant plasmid. **B.** The phosphorylation of wild-type of CSF1R was activated upon CSF1 treatment whereas the phosphorylation of CSFIR-I794T variant was undetectable.
